# Supplementary material for: Mutations in PCYT2 disrupt etherlipid biosynthesis and cause a complex hereditary spastic paraplegia
Source: Brain. 2019 Oct 22;142(11):3382–97. doi: 10.1093/brain/awz291 (PMC6821184; doi:10.1093/brain/awz291)
Supplement: awz291_Supplementary_Data [file awz291_supplementary_data.zip › awz291-Suppl_data/Supplementary_Data4.zip!/Supplementary information 4 - plasma lipidomics.zip!/Plasma lipidomics/Control vs Patient3b/Statistical plots and data/Volcano plot_alternative.pdf]

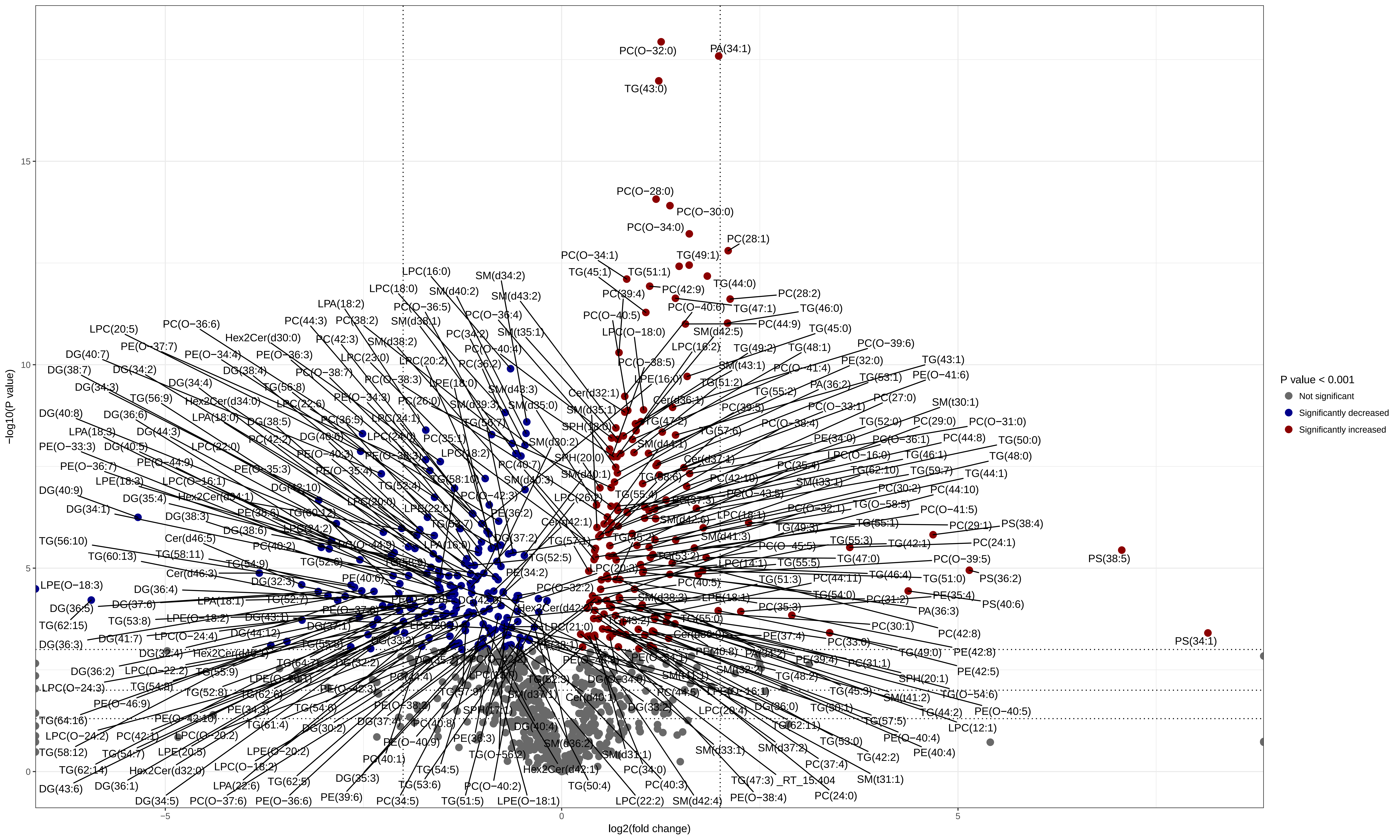

P value < 0.001
Not significant
Significantly decreased
Significantly increased
−log10(P value)
log2(fold change)
15
10
5
0
−5
0
5
PC(O−32:0)
PA(34:1)
TG(43:0)
PC(O−28:0)
PC(O−30:0)
PC(O−34:0)
PC(28:1)
PC(O−34:1)
TG(49:1)
TG(45:1)
TG(51:1)
TG(44:0)
PC(39:4)
PC(42:9)
PC(28:2)
PC(O−40:5)
PC(O−40:6)
TG(47:1)
TG(46:0)
LPC(O−18:0)
PC(44:9)
TG(45:0)
SM(d42:5)
LPC(16:2)
TG(49:2)
TG(48:1)
PC(O−39:6)
PC(O−38:5)
SM(t43:1)
PE(32:0)
TG(43:1)
LPE(16:0)
PC(O−41:4)
TG(53:1)
PE(O−41:6)
TG(51:2)
PA(36:2)
Cer(d32:1)
TG(55:2)
PC(27:0)
SM(t30:1)
Cer(d36:1)
PC(39:5)
PC(O−33:1)
SM(d35:1)
TG(47:2)
PC(O−38:4)
TG(52:0)
PC(29:0)
PC(O−31:0)
SPH(18:0)
TG(57:6)
PE(34:0)
PC(O−36:1)
PC(44:8)
TG(50:0)
SM(d44:1)
LPC(O−16:0)
TG(46:1)
TG(48:0)
Cer(d37:1)
PC(35:4)
TG(62:10)
TG(59:7)
TG(44:1)
TG(58:6)
PC(42:10)
SM(t33:1)
PC(30:2)
PC(44:10)
TG(55:4)
PC(O−43:5)
PE(37:3)
TG(O−58:5)
PC(O−32:1)
PC(O−41:5)
SM(d42:6)
LPC(18:1)
TG(55:1)
PC(29:1)
PS(38:4)
TG(49:3)
TG(45:2)
SM(d41:3)
TG(55:3)
TG(42:1)
PC(24:1)
PC(O−45:5)
TG(53:2)
TG(47:0)
PS(38:5)
LPC(20:3)
LPC(14:1)
TG(55:5)
PC(O−39:5)
TG(51:3)
PC(44:11)
TG(46:4)
TG(51:0)
PS(36:2)
PC(40:5)
SM(d38:2)
LPE(18:1)
TG(54:0)
PC(31:2)
PE(35:4)
PA(36:3)
PS(40:6)
PC(35:3)
TG(55:0)
PC(30:1)
PC(42:8)
PS(34:1)
TG(43:2)
Cer(d36:0)
PE(37:4)
PC(33:0)
PE(42:8)
TG(49:0)
PE(40:8)
PA(34:2)
PE(39:4)
PC(31:1)
PE(42:5)
SPH(20:1)
TG(48:2)
SM(t41:2)
TG(O−54:6)
TG(45:3)
PC(44:5)
LPE(O−16:1)
TG(44:2)
PE(O−40:5)
DG(36:0)
TG(50:1)
LPC(12:1)
LPC(20:4)
TG(57:5)
TG(62:11)
PE(O−40:4)
TG(53:0)
PE(40:4)
SM(d33:1)
SM(d37:2)
PC(37:4)
TG(42:2)
SM(t31:1)
TG(47:3)_RT_15.404
PC(24:0)
PE(O−38:4)
SM(d42:4)
PC(40:3)
LPC(22:2)
PC(34:0)
TG(50:4)
SM(d31:1)
Hex2Cer(d42:1)
LPE(O−18:1)
TG(51:5)
PC(O−40:2)
PC(34:5)
TG(53:6)
TG(54:5)
PE(39:6)
DG(35:3)
PE(O−36:6)
PC(O−37:6)
DG(34:5)
LPA(22:6)
TG(62:5)
LPC(O−18:2)
Hex2Cer(d32:0)
DG(36:1)
DG(43:6)
TG(62:14)
TG(58:12)
TG(54:7)
LPE(20:5)
LPE(O−20:2)
PC(40:1)
PE(O−40:9)
PE(38:3)
TG(O−56:2)
SM(d36:2)
DG(40:4)
LPC(O−24:2)
PC(42:1)
LPC(O−20:2)
TG(64:16)
PE(O−42:10)
TG(61:4)
DG(30:2)
DG(37:4)
PC(40:8)
SPH(17:1)
PE(O−46:9)
PE(34:3)
TG(54:6)
PE(O−38:2)
Cer(d40:1)
DG(33:2)
LPC(O−24:3)
TG(54:8)
TG(52:8)
TG(62:6)
PE(O−42:3)
TG(57:9)
SM(d37:1)
DG(O−34:0)
DG(36:2)
LPC(O−22:2)
TG(55:9)
LPE(O−20:1)
PC(44:4)
LPC(16:0)
SM(d34:2)
LPC(18:0)
SM(d40:2)
SM(d43:2)
LPA(18:2)
PC(O−36:5)
PC(O−36:4)
PC(44:3)
PC(38:2)
SM(d38:1)
LPC(20:5)
PC(O−36:6)
SM(t35:1)
PC(34:2)
Hex2Cer(d30:0)
PC(42:3)
SM(d38:2)
PE(O−37:7)
PC(O−40:4)
DG(40:7)
PE(O−34:4)
PE(O−36:3)
LPC(23:0)
LPC(20:2)
PC(36:2)
DG(38:7)
DG(34:2)
DG(38:4)
PC(O−38:7)
PC(O−38:3)
DG(34:4)
TG(56:8)
LPE(18:0)
SM(d43:3)
DG(34:3)
TG(56:9)
PE(O−34:3)
Hex2Cer(d34:0)
LPC(22:6)
PC(26:0)
SM(d39:3)
SM(d35:0)
DG(40:8)
DG(36:6)
LPA(18:0)
DG(38:5)
PC(36:5)
LPC(24:1)
TG(56:7)
LPA(18:3)
DG(44:3)
PC(42:2)
DG(40:6)
LPC(24:0)
PC(35:1)
SM(d30:2)
PE(O−33:3)
DG(40:5)
LPC(22:0)
PE(O−40:3)
PE(O−38:3)
LPC(18:2)
SPH(20:0)
PE(O−44:9)
PC(40:7)
PE(O−36:7)
PE(O−35:3)
PE(O−35:4)
SM(d40:1)
LPE(18:3)
LPC(O−16:1)
TG(58:10)
SM(d40:3)
DG(40:9)
DG(42:10)
TG(52:4)
DG(35:4)
Hex2Cer(d34:1)
PC(O−42:3)
LPC(26:2)
LPC(20:0)
DG(34:1)
PE(38:6)
TG(60:12)
LPE(22:6)
PE(36:2)
DG(38:3)
TG(53:7)
Cer(d42:1)
LPC(24:2)
DG(38:6)
DG(37:2)
Cer(d46:5)
TG(57:1)
TG(56:10)
PC(40:2)
PC(O−44:9)
LPA(16:0)
TG(58:11)
TG(60:13)
TG(52:5)
TG(54:9)
TG(52:6)
TG(58:9)
Cer(d46:3)
PE(34:2)
PE(40:6)
LPE(O−18:3)
DG(32:3)
DG(36:4)
PC(O−32:2)
DG(42:8)
DG(36:5)
LPA(18:1)
TG(52:7)
DG(37:6)
Hex2Cer(d42:2)
PE(O−37:6)
TG(53:8)
LPE(O−18:2)
DG(43:1)
TG(62:15)
DG(37:1)
LPC(20:1)
LPC(21:0)
DG(41:7)
LPC(O−24:4)
DG(44:12)
TG(33:3)
DG(36:3)
TG(55:8)
PE(38:1)
DG(32:4)
Hex2Cer(d40:1)
DG(35:2)
PC(O−42:2)
PE(O−43:8)
PE(O−34:1)
SM(d32:2)
TG(64:7)
DG(32:2)
TG(52:9)
SM(t41:1)
